# The role and effect of companions during childbirth in Oman

**DOI:** 10.1186/s12884-024-06256-x

**Published:** 2024-01-09

**Authors:** Nasar Alwahaibi, Rodina AL-Julandani, Alzarah Al-Kalbani

**Affiliations:** Department of Biomedical Science, College of Medicine and Health Sciences, Sultan Qaboos University, P.O. Box 35, Muscat, 123 Oman

**Keywords:** Companion, Childbirth, Labour, Pregnant women, Oman

## Abstract

**Background:**

There is increasing evidence that a companionship is an important tool for improving the quality of care provided to pregnant women during the labour and delivery process. The literature review shows very limited studies assessing the role of companions during childbirth from the companion’s point of view. Many published papers studied the role and satisfaction of pregnant women but not their companions. Therefore, this study aimed to assess the role and effect of companions during childbirth in Oman.

**Methods:**

This cross-sectional observational study was conducted at Sultan Qaboos University Hospital, Oman, between June 2022 and April 2023. Companions were interviewed face-to-face at a time convenient to them. A telephone interview was conducted with those who left the hospital early. The questionnaire comprised sociodemographic data and other sections, including the nature of the help provided by the supportive companion, their effects on the women who gave birth, and the timing of their presence during companionship.

**Results:**

A total of 214 companions were included in this study with the mean age of 42.54 years. The most common relationship to the pregnant women was mothers (35.7%), followed by husbands (30.5%). The majority of companions provided support during admission (62.6%), in the immediate post-partum ward (56.5%) and during delivery (54.2%), while a minority helped from admission to discharge (22.4%). The most common type of support provided was encouraging words (89.7%) followed by transferring things (43.9%), massage (37.4%) and touch (33.6%). The majority of companions (96.7%) reported that their support helped very much, and the pregnant women felt better and calmer.

**Conclusions:**

Labouring women felt better and calmer because of the presence of companions. Companions preferred to be present in the postpartum and during labour and delivery. The majority of companions support their labouring women by encouraging wards. Companions love and encourage others to support their labouring women during their critical times.

## Background

There is a well-defined concept of labour support, which essentially refers to the provision of information, advice, and comfort measures to help a woman cope well with the rigors of labour and birth [1]. Birth companions in Western society are associated with positive birth outcomes and greater control over the labour process [2]. However, these support people are different from those commonly available and used in developing countries [1, 2].

There is increasing evidence that a companionship is an important tool for improving the quality of care provided to pregnant women during the labour and delivery process, and the World Health Organization (WHO) has confirmed the importance of companionship for pregnant women [3]. Because of the support provided by the companion, the labouring experience is easier for the women. The pain can be reduced because of the companion’s presence. According to a study conducted in 2011, companionship during labour has a positive effect on both mother and baby [4]. In addition, the presence of companions during labour, makes the women feel safe and comfortable, positively influences women`s psychological health including comforting touch, improves maternal-infant bonding, and reduces anxiety and depression [5]. In fact, it improves women`s physiological status because the women who have support will be less likely to have a caesarean section, intrapartum analgesia, and labour more likely to be shorter [6]. On the other hand, women without support may experience longer labour and feel exhausted [7].

Globally, men are encouraged to attend their children’s births, which helps them better understand the birth process by being involved in the preparations. As a result, they feel more involved in labour and can make birth decisions together with their spouses [8]. It also promotes the feeling of gratitude, as well as makes the father feel satisfied and emotional to become a father. In contrast, some fathers may experience negative effects during childbirth when present. Fathers may feel compelled to play an active role during labour and delivery, which may leave them feeling helpless and useless, especially when they have to witness the pain of their partners [8].

It is the first study that has focused on the role and effect of the companions during childbirth from the perspective of the companion during the birth process. We mean by the role is the nature of the help provided by the companions during childbirth such as massage, touch, encouraging words, and transfer things. Whereas the effect is the outcome of the companion’s presence such as helped bit, very much helped, or didn’t notice any difference. Despite the fact that a great deal of published research has focused on pregnant women’s role and satisfaction, very few studies have looked at their companions [5, 6]. Furthermore, studies related to the involvement of Omani husbands/mothers/sisters during childbirth are also lacking despite the high birth rates due to community characteristics of a high desire to have large families. Therefore, the aim of this study was to assess the role and effect of companions during childbirth.

## Methods

### Study design

This cross-sectional observational study was conducted between June 2022 and April 2023. The study was conducted in the Obstetric ward at Sultan Qaboos University Hospital, Oman. This hospital is a governmental referral university hospital with about 3274 Omani deliveries per year (data based on the last five years). It has an average of 273 births per month. Oman is a country in Western Asia. It is situated on the southeastern coast of the Arabian Peninsula, covers country area of 309,500 sq km, and by 2023 Oman population is estimated to be 5,5 million [9]. Oman comprises 11 governorates: Muscat, Buraymi, the Dakhiliyah, the North Batinah, the South Batinah, the South Sharqiyah, the North Sharqiyah, the Dhahirah, Dhofar, Musandam, and the Wusta. The inclusion criteria include companions of various ages, genders, and degrees of compatibility (mother, father, sister, husband, friend, etc.). As well as the companions with whom the pregnant women delivered a live baby. The exclusion criteria include companions who struggle to communicate because of disease or any other condition, as well as those who refuse to be interviewed. During the study, data were collected based on the availability of researchers. Additionally, the questionnaire was available in both English and Arabic versions. Companions were interviewed face-to-face at a time convenient to them. The place was private and all answers were kept unidentified and confidential. A telephone interview was conducted with those who left the hospital early.

### Sample size calculation

The sample size for the study was determined using the prevalence rate of 13.3% found in other study, which was carried out in Al-Ain, United Arab Emirates [10]. Using the formula N = p(1 − p) z^2^/d^2^ [11], 95% confidence interval, 5% margin of error, and 20% non-response rate (to avoid any incorrectly filled-out questionnaires), the sample size was 214. A pilot study was conducted among 15 companions who fulfilled the research criteria. Those who participated in the pilot study were excluded from the study. In addition, five experts reviewed the questionnaire. Some questions were subsequently modified to be understandable. The Cronbach’s alpha for the reliability of the questionnaire was 0.704.

### Data collection

The questionnaire consisted of two sections. The first section was about sociodemographic information such as age, residence, relative degree, educational level, and occupation. The second section was about the nature of the help provided by the supportive companion (touch, massage, encouraging wards, or transfer things) if the companion faced any problem during providing the help and what was the reason, their effects on the women who gave birth and if their presence as a supportive companion had a positive or negative effect. Moreover, companions asked if they would present as supportive companions again and if they encourage others to be a companion. As well as about the companion`s satisfaction degree about the medical team’s efforts and services provided to the companions. This Questionnaire contains a number of self-developed questions as well as some other questions obtained from literature reviews [6, 10]. Type 1 includes only one support either touch only, massage only, encouraging words only, or transfer things only. Type 2 includes any two supports. Type 3 includes any three supports whereas type 4 includes all the four supports.

### Data analysis

Data was analysed using IBM SPSS Statistics 29.0 (IBM Corp. Released 2022. IBM SPSS Statistics for Windows, Version 28.0. Armonk, NY: IBM Corp). For the descriptive purposes, categorical data was presented using frequency and percentages, and continuous data was presented using mean with standard deviation. Associations between categorical variables were assessed using Chi-square test, while associations between continuous and categorical variables were tested using ANOVA test or independent ‘t’ test depending on the nature of the categorical variables. A *P*-value of < 0.05 was considered as statistical significance.

### Ethical consideration

The Medical Research Ethics Committee (MREC), College of Medicine and Health Sciences, Sultan Qaboos University (SQU), Oman, approved this study, with an ethical approval number SQU- MREC #2776. Preceding the study, a detailed procedure of the study was explained, and each companion signed an informed consent. For illiterates, witnesses who are not part of the research team, were requested to witness the entire process and sign the informed consent. It was made clear to all companions that they can withdraw from the study at any time and would not be asked any questions about why they no longer want to take part. In addition, the medical services presented to the pregnant women and companions would not be affected if they withdraw.

## Results

A total of 214 companions were included in this study. The mean age of the companions was 42.54 ± 9.76 years. The majority of them were from South Batinah (35.0%), followed by Muscat (32.2%). The most common relationship to the pregnant women was being a mother (35.7%), followed by husband (30.5%). The most common education level was secondary (31.8%), followed by primary (10.7%) and 18.2% were illiterates. Most of them were housewife (for women companions) (47.2%), followed by working (44.4%). The majority of participants have their own car (62.6%), 56% of them have own income. The majority of them did not employ a housemaid (61.2%). Diabetes was present in 14.0% of the participants, and hypertension was present in 15.4%. Almost all companions (86.0%) occupied a common room in the labour ward, while a minority (14.0%) occupied a private room (Table 1).

**Table 1:** Socio-demographic characteristics of companions in Sultan Qaboos University Hospital, Oman, 2023 (*n* = 214)

Variable	Frequency	Percentage
Age in years (Mean ± SD)	42.54 ± 9.76	
**Region**		
South Batinah	75	35.0
Muscat	69	32.2
Dhakiliyah	34	15.9
North Batinah	20	9.3
North sharqiya	8	3.7
Ad Dhahira	4	1.9
South sharqiya	3	1.4
Buraimi	1	0.5
**Relationship**		
Mother	76	35.7
Husband	65	30.5
Sister	39	18.3
Aunt	14	6.6
Others	19	8.9
**Education**		
Illiterate	39	18.2
Preparatory	20	9.3
Primary	23	10.7
Secondary	68	31.8
UG	49	22.9
PG	15	7.0
**Occupation**		
Housewife	101	47.2
Working	95	44.4
Not working / Retired	18	8.4
**Own car**		
No	80	37.4
Yes	134	62.6
**Own income**		
No	94	43.9
Yes	120	56.1
**Housemaid**		
No	131	61.2
Yes	83	38.8
**Diabetic**		
No	184	86.0
Yes	30	14.0
**Hypertensive**		
No	181	84.6
Yes	33	15.4
**Type of room**		
Common	184	86.0
Private	30	14.0

UG: undergraduate, PG: postgraduate

The majority of companions provided support during admission (62.6%), in the immediate post-partum ward (56.5%) and during delivery (54.2%, while a minority helped from admission to discharge (22.4%), The most common type of support provided was encouraging words (89.7%) followed by transferring things (43.9%), massage (37.4%) and touch (33.6%). The majority of companions (96.7%) reported that their support helped very much, and only 1.9% and 1.4% of companions reported that they didn’t notice any effect or noticed little effect, respectively. The vast majority of companions (95.3%) said that they would be a companion again, and that they would encourage others to be companions during birth (97.2%). The vast majority of companions (94.9%) were satisfied with the medical team, and 40.7% were satisfied with the services provided by the hospital. Few of them faced problems while helping (10.7%) (Table 2).

**Table 2:** Descriptive for the support outcome variables of companions in Sultan Qaboos University Hospital, Oman, 2023 (*n* = 214)

Variable	Frequency	Percentage
**When did your help take place?**		
During admission	134	62.6
From admission to discharge	48	22.4
Immediate post-partum ward	121	56.5
During delivery	116	54.2
**What is the nature of your help?**		
Massage	80	37.4
Touch	72	33.6
Encouraging words	192	89.7
Transfer things	94	43.9
**Scope of supports**		
1 type of support	98	45.8
2 types	47	22.0
3 types	25	11.7
4 types	44	20.6
**Did you face any problem in helping?**		
No	191	89.3
Yes	23	10.7
**What is the effect of your presence as companion?**		
Helped bit	3	1.4
Very much helped	207	96.7
I didn’t notice any difference	4	1.9
**Perceived effect on the patients**		
**No effects**	**4**	**1.9**
**Little effects**	**3**	**1.4**
Good effects	207	96.7
**Will you be a companion again?**		
No	10	4.7
Yes	204	95.3
**Do you encourage others to be a companion during birth?**		
No	6	2.8
Yes	208	97.2
**Were you satisfied with the medical team?**		
No	11	5.1
Yes	203	94.9
**Were you satisfied with services at hospital?**		
No	127	59.3
Yes	87	40.7

In order to see the associations between variables and the scope of provided support, we categorized each support to four types as detailed in Table 3. 20.6% of companions provided all four types of help to the pregnant women, while majority (45.8%) provided one type of support.

**Table 3:** Categorization of scope of support of companions in Sultan Qaboos University Hospital, Oman, 2023 (*n* = 214)

Scope of support	Categories	Frequency	Percentage
One type of support	Massage only	6	2.8
	Touch only	-	-
	Encouraging words only	79	36.9
	Transfer things only	13	6.1
Two types of support	Massage + Touch	-	-
	Massage + Encouraging words	8	3.7
	Touch + Encouraging words	6	2.8
	Encouraging words + Transfer things	33	15.4
Three types of support	Massage + Touch + Encouraging words	20	9.3
	Massage + Encouraging words + Transfer things	3	1.4
	Massage + Touch + Transfer things	-	-
	Touch + Encouraging words + Transfer things	2	0.9
Four types of support	Massage + Touch + Encouraging words + Transfer things	44	20.6

With regard to the association testing between various factors and the scope of provided support, there was no statistically significant difference observed between the scope of provided support and age, regions, education level, occupation, whether they own a car, own income, having housemaid, and whether they are diabetic or hypertensive. The type of room that they selected for the hospital services did not show statistically significant association (*P* = 0.387). However, age in years showed nearly significant association with the scope of provided support (*P* = 0.056). With regard to relationship, it was observed that maximum help was provided by aunt and husbands as 35.7% and 27.7% of them provided all the four types of help to their patients, respectively, and the least support providers were sisters and others. However, this was not significant (Table 4).

**Table 4:** Association between variables and scope of support provided for companions in Sultan Qaboos University Hospital, Oman, 2023 (*n* = 214)

Variable	Scope of support				*P*-Value
	1 type (*n* = 98)	2 types (*n* = 47)	3 types (*n* = 25)	4 types (*n* = 44)	
Age in years (Mean ± SD)	43.02 ± 10.07	41.40 ± 9.69	46.64 ± 9.42	40.34 ± 8.76	0.056
**Region**					
South Batinah	41 (54.7)	12 (16.0)	7 (9.3)	15 (20.0)	0.370
Muscat	29 (42.0)	15 (21.7)	11 (15.9)	14 (20.3)	
Dhakiliyah	15 (44.1)	7 (20.6)	4 (11.8)	8 (23.5)	
North Batinah	4 (20.0)	10 (50.0)	2 (10.0)	4 (20.0)	
North sharqiya	4 (50.0)	2 (25.0)	1 (12.5)	1 (12.5)	
Ad Dhahira	2 (50.0)	1 (25.0)	-	1 (25.0)	
South sharqiya	3 (100.0)	-	-	-	
Buraimi	-	-	-	1 (100.0)	
**Relationship**					
Mother	41 (53.9)	13 (17.1)	9 (11.8)	13 (17.1)	0.086
Husband	31 (47.7)	13 (20.0)	3 (4.6)	18 (27.7)	
Sister	14 (35.9)	13 (33.3)	6 (15.4)	6 (15.4)	
Aunt	3 (21.4)	4 (28.6)	2 (14.3)	5 (35.7)	
Others	8 (42.1)	4 (21.1)	5 (26.3)	2 (10.5)	
**Education**					
Illiterate	21 (53.8)	7 (17.9)	5 (12.8)	6 (15.4)	0.995
Preparatory	9 (45.0)	5 (25.0)	2 (10.0)	4 (20.0)	
Primary	11 (47.8)	5 (21.7)	4 (17.4)	3 (13.0)	
Secondary	29 (42.6)	17 (25.0)	7 (10.3)	15 (22.1)	
UG	21 (42.9)	10 (20.4)	6 (12.2)	12 (24.5)	
PG	7 (46.7)	3 (20.0)	1 (6.7)	4 (26.7)	
**Occupation**					
Housewife	49 (48.5)	18 (17.8)	16 (15.8)	18 (17.8)	0.108
Working	43 (45.3)	22 (23.2)	6 (6.3)	24 (25.3)	
Not working / Retired	6 (33.3)	7 (38.9)	3 (16.7)	2 (11.1)	
**Own car**					
No	36 (45.0)	23 (28.7)	8 (10.0)	13 (16.3)	0.248
Yes	62 (46.3)	26 (19.4)	17 (12.7)	31 (23.1)	
**Own income**					
No	42 (44.7)	24 (25.5)	10 (10.6)	18 (19.1)	0.722
Yes	56 (46.7)	23 (19.2)	15 (12.5)	26 (21.7)	
**Housemaid**					
No	54 (41.2)	33 (25.2)	17 (13.0)	27 (20.6)	0.299
Yes	44 (53.0)	14 (16.9)	8 (9.6)	17 (20.5)	
**Diabetic**					
No	86 (46.7)	38 (20.7)	22 (12.0)	38 (20.7)	0.712
Yes	12 (40.0)	9 (30.0)	3 (10.0)	6 (20.0)	
**Hypertensive**					
No	86 (47.5)	40 (22.1)	22 (12.2)	33 (18.2)	0.252
Yes	12 (36.4)	7 (21.2)	3 (9.1)	11 (33.3)	
**Type of room**					
In the labor ward	87 (47.3)	37 (20.1)	21 (11.4)	39 (21.2)	0.387
Private	11 (36.7)	10 (33.3)	4 (13.3)	5 (16.7)	

UG: undergraduate, PG: postgraduate

Providing the four types of support in the post-partum ward was significantly lower compared to other times (*P* = 0.004). All other characteristics related to the provided support did not show any statistically significant association with the scope of support. Interestingly, those who were not satisfied with hospital services provided larger scope of support to the pregnant women compared to those who were more satisfied, however this was not significant (Table 5).

**Table 5:** Association between support characteristics and scope of provided support of companions in Sultan Qaboos University Hospital, Oman, 2023 (*n* = 214)

Variables	Scope of support				*P*-Value
	1 type (*n* = 98)	2 types (*n* = 47)	3 types (*n* = 25)	4 types (*n* = 44)	
**When did your help take place?**					
During admission	64 (47.8)	26 (19.4)	17 (12.7)	27 (20.1)	0.634
From admission to discharge	16 (33.3)	14 (29.2)	5 (10.4)	13 (27.1)	0.177
Immediate post-partum ward	68 (56.2)	23 (19.0)	9 (7.4)	21 (17.4)	0.004
During delivery	59 (50.9)	23 (19.8)	10 (8.6)	24 (20.7)	0.264
**Did you face any problem in helping?**					
No	88 (46.1)	40 (20.9)	23 (12.0)	40 (20.9)	0.768
Yes	10 (43.5)	7 (30.4)	2 (8.7)	4 (17.4)	
**Perceived effect**					
No/Little effect Good effect	5 (71.4) 93 (44.9)	2 (25.6) 45 (21.7)	- 25 (12.1)	- 44 (21.3)	0.132
**Will you be a companion again?**					
No	8 (80.0)	1 (10.0)	-	1 (10.0)	0.104
Yes	90 (44.1)	46 (22.5)	23 (11.3)	43 (21.1)	
**Do you encourage others to be a companion during birth?**					
No	4 (66.7)	1 (16.7)	-	1 (16.7)	0.557
Yes	94 (45.2)	46 (22.1)	25 (12.0)	43 (20.7)	
**Were you satisfied with the medical team?**					
No	4 (36.4)	4 (36.4)	-	3 (27.3)	0.258
Yes	94 (46.3)	43 (21.2)	25 (12.3)	41 (20.2)	
**Were you satisfied with services at hospital?**					
No	53 (41.7)	25 (19.7)	18 (14.2)	31 (24.4)	0.123
Yes	45 (51.7)	22 (25.3)	7 (8.0)	13 (14.9)	

We combined the very much helped (she felt better and calmer) as a good effect and helped bit and/or I didn’t notice any difference as no or little effect, and compared with other sociodemographic factors. The results showed that the husbands of the pregnant women were less likely to score the effect as good compared to mothers and other relatives (*P*-value = 0.028). In addition, who owned a car were more likely (99.3%) to score the effect as good than those who did not own a car (92.5%, *P*-value = 0.012). The other variables including age, region, education, occupation, own income, having housemaid, being diabetic, hypertension, and type of room did not show a statistically significant association with the perceived effect (Table 6).

**Table 6:** Association between factors and the perceived effect of the provided support of companions in Sultan Qaboos University Hospital, Oman, 2023 (*n* = 214)

Variables	Perceived effect		*P*-Value
	No/Little (*n* = 7)	Good (*n* = 207)	
Age in years (Mean ± SD)	36.57 ± 7.44	42.74 ± 9.78	0.100
**Region**			
South Batinah	-	75 (100.0)	0.081
Muscat	4 (5.8)	65 (94.2)	
Dhakiliyah	-	34 (100.0)	
North Batinah	1 (5.0)	19 (95.0)	
North sharqiya	1 (12.5)	7 (87.5)	
Ad Dhahira	1 (25.0)	3 (75.0)	
South sharqiya	-	3 (100.0)	
Buraimi	-	1 (100.0)	
**Relationship**			
Mother	1 (1.3)	75 (98.7)	0.028
Husband	6 (9.2)	59 (90.8)	
Sister	-	39 (100.0)	
Aunt	-	14 (100.0)	
Others	-	19 (100.0)	
**Education**			
Illiterate	-	39 (100.0)	0.255
Preparatory	1 (5.0)	19 (95.0)	
Primary	-	23 (100.0)	
Secondary	4 (5.9)	64 (94.1)	
UG	2 (4.1)	47 (95.9)	
PG	-	15 (100.0)	
**Occupation**			
Housewife	1 (1.0)	100 (99.0)	0.059
Working	6 (6.3)	89 (93.7)	
Not working / Retired	-	18 (100.0)	
**Own car**			
No	6 (7.5)	74 (92.5)	0.012
Yes	1 (0.7)	133 (99.3)	
**Own income**			
No	4 (4.3)	90 (95.7)	0.702
Yes	3 (2.5)	117 (97.5)	
**Housemaid**			
No	5 (3.8)	126 (96.2)	0.709
Yes	2 (2.4)	81 (97.6)	
**Diabetic**			
No	5 (2.7)	179 (97.3)	0.255
Yes	2 (6.7)	28 (93.3)	
**Hypertensive**			
No	5 (2.8)	176 (97.2)	0.295
Yes	2 (6.1)	31 (93.9)	
**Type of room**			
Common	7 (3.8)	177 (96.2)	0.597
Private	-	30 (100.0)	

UG: undergraduate, PG: postgraduate

In addition, we explored if support related characteristics can affect the perceived effect. There was no statistically significant association found between the reported perceived effect and: timing of provided support, the nature of support provided, the scope of supports provided, the difficulties during support delivery, willing to be a companion again or not, satisfaction with medical team and satisfaction with hospital services. However, there was a significant difference found between those who encourage others to be a companion during birth and those who do not. In this regard, 97.6% of encouragers perceived their support to have good effect, compared to 66.7% of non-encouragers (*P* = 0.013) (Table 7).

**Table 7:** Association between support related characteristics and perceived effect of the provided support of companions in Sultan Qaboos University Hospital, Oman, 2023 (*n* = 214)

Variables	Perceived support		*P*-Value
	No/Little (*n* = 7)	Good (*n* = 207)	
**When did your help take place?**			
During admission	5 (3.7)	129 (96.3)	1.000
From admission to discharge	1 (2.1)	47 (97.9)	1.000
Immediate post-partum ward	6 (5.0)	115 (95.0)	0.141
During delivery	5 (4.3)	111 (95.7)	0.457
**What is the nature of your help?**			
Massage	1 (1.3)	79 (98.8)	0.261
Touch	-	72 (100.0)	0.098
Encouraging words	5 (2.6)	187 (97.4)	0.154
Transfer things	3 (3.2)	91 (96.8)	1.000
**Scope of supports**			
1	5 (5.1)	94 (94.9)	0.149
2	2 (4.1)	47 (95.9)	
3	-	23 (100.0)	
4	-	43 (100.0)	
**Did you face any problem in helping?**			
No	7 (3.7)	184 (96.3)	1.000
Yes	-	23 (100.0)	
**Will you be a companion again?**			
No	1 (10.0)	9 (90.0)	0.288
Yes	6 (2.9)	198 (97.1)	
**Do you encourage others to be a companion during birth?**			
No	2 (33.3)	4 (66.7)	0.013
Yes	5 (2.4)	203 (97.6)	
**Were you satisfied with the medical team?**			
No	-	11 (100.0)	1.000
Yes	7 (3.4)	196 (96.6)	
**Were you satisfied with services at hospital?**			
No	4 (3.1)	123 (96.9)	1.000
Yes	3 (3.4)	84 (96.6)	

## Discussion

This is the first study, which focused on the role of the companion during childbirth from the companion’s point of view. Unfortunately, many published papers studied the role and satisfaction of pregnant women but not their companions. During these studies, only pregnant women and not their companions were interviewed quantitatively or qualitatively, as they included the companions’ responses [3, 10, 12–14].

According to this study, Omani pregnant women prefer mothers over husbands as companions. Due to the culture of society as the daughters are very close, feel more comfortable, and safe with their mothers. Also, the mothers, who have been through this experience, knew the worries of their daughters. Needless to mention that women share with other women many things that cannot be shared with men. This finding (mothers as companions 35.7%) is in line with another study which reported that mothers as companions represented 34.5% [3].

In addition, a study conducted in the United Arab Emirates reported that 59% of pregnant women had mother companions during childbirth [10]. Surprisingly, a study aimed to describe Saudi women’s preferences toward supportive companions during labour, showed that 54.7% did not prefer the presence of any companion [12]. According to the authors study, this high number may be due to the lack of understanding among surveyed Saudi women about the importance and benefits of having support during childbirth as well as the lack of a standardized policy in most governmental hospitals for allowing a companion to be present during labor. In another recent study, 39% of Saudi women who gave childbirth did not have companions [13]. In addition, in a study of the 70 Russian women interviewed about the presence of a support person during labour, 68.6% declined to have a partner present during labour. According to the respondents, the most common reasons were having a private experience (22.9%), feeling personally embarrassed (17.1%), feeling afraid for their spouse (15.7%), and fearing that it would adversely affect their sexual life (8.6%) [14].

Husbands represented the second favorable companions in this study. Because of their relationship with their wives, they are the second choice for many Omani women. An American study showed that husbands’ presence during childbirth was 92.3% [15]. In the in the United States, during labor and delivery, fathers usually accompany their wives/partners. In addition, mothers wish for their fathers to be present at their babies’ births [15]. In comparison with the current study, Omani women, as mentioned previously, feel very close, more comfortable, and safe with their mothers. The importance of husbands during childbirth has been documented in many studies. Various studies showed that husbands’ presence makes labouring women safer and more secure, reduces maternal stress, and therefore enhances maternal well-being, increases interest in prenatal care, provides psychological support, and women feel more in control [16–19].

The current study showed that the average age of companions is 42.54 years old. In agreement with a Brazilian study, which evaluated companions’ knowledge of the support they can offer during childbirth, the average age of companions who attended the birth was 44.3 years old [5]. The present study shows that 89.3% of companions did not face any problems during their stay in the delivery ward; this means that the medical team welcomed companions without any reservations or conditions.

Despite the recommendations of the WHO to have companions during childbirth even during the coronavirus disease (COVID-19) [20], many hospitals, as a safety precaution, have restricted the presence of companions during childbirth [21]. In fact, not all hospitals allow the presence of companions, in Thailand, where family members usually were not allowed to be present during intrapartum [22]. Others like Ethiopia have a low labour companionship, which was found to be 14.6% [23]. In a recent study in Burkina Faso, where 77 women, companions, and health workers were interviewed about their beliefs, opinions, and policies about labour companions, they found that hospitals were not allowing companions during labour and birth [24].

The present study showed that 89.7% of the companions used encouraging wards as a help to their labouring women. This finding is in line with another study, which showed that 82% of Brazilian companions (52/62) would use encouraging wards as psychological support [5]. Probably encouraging wards are easy and effective for the companions to do during childbirth.

The present study shows that the moments of companionship were high in the postpartum ward (56.5%) and during labour and delivery (54.2%). In the present study, companions include mainly mothers and husbands, and to a lesser extent, sisters and aunts. This might explain why the presence of companions is high in the postpartum as husbands wanted to see their children straight after the delivery and some might be afraid to hear the pain of their wives. Nevertheless, their presence during labour and delivery is still high, which means that there are caring and keen to minimize the pain of their wives. In agreement with the current study, another Brazilian study showed that the moments of companionship were 74.8% during labour and delivery and 61.3% during postpartum [6].

The present study shows that 96.7% of companions noticed that their presence was very helpful, and the women felt better and calmer. A similar finding was observed in a Brazilian study where 91.2% of women considered that having a companion during labour and birth was helpful and had a better and calmer birth experience [6]. In Poland, 37 couples were evaluated for their father’s presence at the delivery ward, the findings showed that women felt better having had their partners with them during labour [25]. In Brazil, 212 women were enrolled in a randomized controlled clinical trial, the results show that the support provided by a companion of the woman’s choice during labour and delivery had a positive effect on her satisfaction with the birth experience [26]. In a randomized control trial for 84 Iranian primiparous women, the researchers found that the presence of trained husbands beside their wives during delivery decreased mothers’ anxiety [27].

Based on the responses of our study, 95.3% of companions were aware of the importance of their presence as supportive companions. Further, 97.2% of them encouraged others to be companions during birth, as companions play an important role in facilitating and making the birth experience less stressful and more positive. In line with the current findings, a Brazilian study, aimed to evaluate the knowledge of companions about the use of support techniques during childbirth, found that among the 62 companions, 95% considered the experience of witnessing the delivery positive [5].

The majority of companions were satisfied with the medical team’s efforts and reported that the team responded quickly to the women’s needs. Since there were no similar studies evaluating the satisfaction of companions during the childbirth, we compared this satisfaction with the pregnant women. In line with our study, the majority of women in Malawi (97.3%) were satisfied with the care they received from admission through labour and delivery [28]. Another study in Ethiopia, revealed that 90.2% of women who gave birth in public health facilities were satisfied with labour and delivery care [29]. In Ethiopia, the proportion of mothers who were satisfied with delivery care was 61.9% [30]. In Iran, the satisfaction level of pregnant women was 59.5% [31]. However, in Jordan, the maternal satisfaction rate was only 17.8% [32]. In our study the medical team was highly rated by companions, but 59.3% of companions expressed dissatisfaction with the obstetric ward services. As far as the food and sleeping mats were concerned, they were not happy.

### Strengths and limitations

A strength of this study is that it is the first to examine the role of companions during childbirth directly rather than through pregnant women. However, the current study has also some limitations. First, women who delivered in private hospitals were not included. Second, the data were collected from one single hospital, even though it is a tertiary teaching hospital with 500 beds. Third, interviews in this study were only conducted with companions whom the pregnant women delivered a live baby.

## Conclusion

Labouring women felt better and calmer because of the presence of companions. Companions preferred to be present in the postpartum and during labour and delivery. The majority of companions support their labouring women by encouraging wards. Companions love and encourage others to support their labouring women during their critical times.

## Data Availability

The datasets used and/or analysed during the current study are available from the corresponding author on reasonable request.

## Abbreviations

WHO: World Health Organization
COVID-19: Coronavirus disease
